# Observation of a quantum Cheshire Cat in a matter-wave interferometer experiment

**DOI:** 10.1038/ncomms5492

**Published:** 2014-07-29

**Authors:** Tobias Denkmayr, Hermann Geppert, Stephan Sponar, Hartmut Lemmel, Alexandre Matzkin, Jeff Tollaksen, Yuji Hasegawa

**Affiliations:** 1 Atominstitut, Vienna University of Technology, Stadionallee 2, 1020 Vienna, Austria; 2 Institut Laue-Langevin, 71 Avenue des Martyrs, CS 20156, 38042 Grenoble Cedex 9, France; 3 Laboratoire de Physique Théorique et Modélisation, CNRS Unité 8089, Université de Cergy-Pontoise, 95302 Cergy-Pontoise, France; 4 Institute for Quantum Studies and Schmid College of Science and Technology, Chapman University, 1 University Drive, Orange, California 92866, USA

## Abstract

From its very beginning, quantum theory has been revealing extraordinary and counter-intuitive phenomena, such as wave-particle duality, Schrödinger cats and quantum non-locality. Another paradoxical phenomenon found within the framework of quantum mechanics is the ‘quantum Cheshire Cat’: if a quantum system is subject to a certain pre- and postselection, it can behave as if a particle and its property are spatially separated. It has been suggested to employ weak measurements in order to explore the Cheshire Cat’s nature. Here we report an experiment in which we send neutrons through a perfect silicon crystal interferometer and perform weak measurements to probe the location of the particle and its magnetic moment. The experimental results suggest that the system behaves as if the neutrons go through one beam path, while their magnetic moment travels along the other.

## Abstract (web summary)

One of the paradoxical phenomena of quantum mechanics is the quantum Cheshire Cat, consisting of the apparent spatial separation of a particle and one of its properties. Denkmayr *et al.* use neutron interferometry to prepare and evaluate the Cheshire Cat state of a neutron and its magnetic moment.

## Graphical abstract

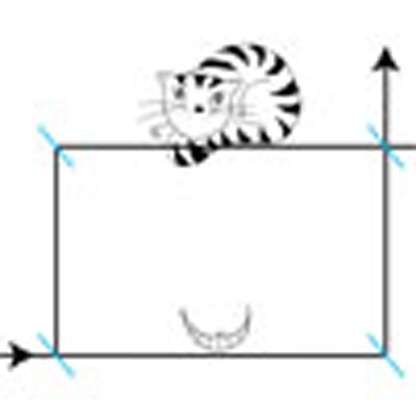

The study of fundamental quantum mechanical phenomena is not only enriching our scientific knowledge, but also our understanding of the natural laws. This understanding lead to the development of numerous technological applications: quantum non-locality^1^,^2^,^3^,^4^ plays an essential role in quantum cryptology^5^, the understanding of wave-particle duality^6^ made semiconductor technology possible^7^ and the investigation of Schrödinger cats^8^ advanced the field of quantum information processing and communication^9^.

During the examination of the quantum measurement process, Aharonov, Albert and Vaidman^10^,^11^ introduced the weak value, defined as


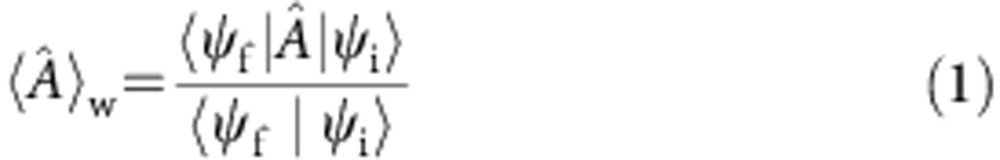


where 
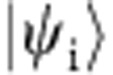
 and 
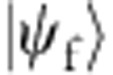
 are the initial (‘preselected’) and final (‘postselected’) states of the system and *Â* is an observable of the system. ‹*Â*›_w_ represents information of the observable *Â* between pre- and postselection, which can be obtained by weakly coupling the system to a measurement device, that is, a probe, without significantly altering the subsequent evolution of the system. Due to the weakness of the coupling between the system and the measurement device, the information obtained by a single measurement is limited. To attain useful information about a quantum system, the measurement has to be repeated several times. The weak value ‹*Â*›_w_ then reflects the average conditioned on the pre- and postselected ensemble^12^.

The first experimental determination of a weak value showed that the weak measurement scheme can be used as a means of amplification.^13^ Subsequently, the experimental work on weak measurements demonstrated that they allow information about a quantum system to be obtained with minimal disturbance^14^,^15^, that they can be used for high-precision metrology^16^,^17^ and that they are perfectly suited for the study of quantum paradoxes^18^,^19^,^20^,^21^,^22^.

A surprising effect originating from pre- and postselection of a system is the ability to ‘separate’ the location of a system from one of its properties^23^,^24^,^25^,^26^ as suggested by the Cheshire Cat story in Alice in Wonderland: ‘Well! I’ve often seen a cat without a grin,’ thought Alice; ‘but a grin without a cat! It’s the most curious thing I ever saw in all my life!’^27^. The essential property of a quantum Cheshire Cat in a Mach–Zehnder-type interferometer is, that the cat itself is located in one beam path, while its grin is located in the other one^25^. An artistic depiction of this behaviour is shown in Fig. 1.

In this work, we prepare and measure the Cheshire Cat states by means of neutron interferometry^28^, which has already been successfully used to study many other purely quantum mechanical effects^29^,^30^,^31^. The experimental results suggest that the system behaves as if the neutrons go through one beam path, while their magnetic moment travels along the other.

## Results

### Theoretical considerations

In our experiment, the neutron plays the role of the cat and the cat’s grin is represented by the neutron’s spin component along the *z* direction. The system is initially prepared so that after entering the beam splitter its quantum state is given by


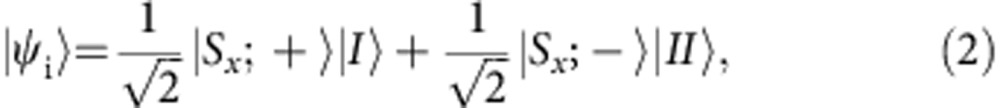


where |*I*› (|*II*›) stands for the spatial part of the wavefunction along path *I* (path *II*) of the interferometer and |*S*_*x*_;±› denotes the spin state in ±*x* direction. In order to observe the quantum Cheshire Cat, after we preselect the ensemble, we will next perform weak measurements of the neutrons’ population in a given path on the one hand and of the value of the spin in a given path on the other. Subsequently, the ensemble is postselected in the final state:


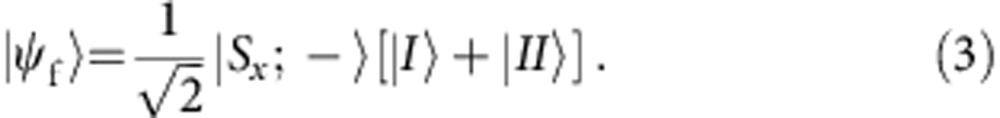


Whenever the postselection succeeds, that is, when a Cheshire Cat is created, a minimally disturbing measurement will find the Cat in the upper beam path, while its grin will be found in the lower one.

This paradoxical behaviour can be quantified using the weak value. We can calculate the weak values of the projection operators on the neutron path eigenstates 
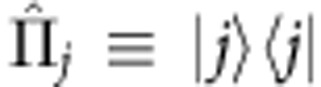
, with *j*=*I*, *II*. From equations (1, 2, 3), we obtain 
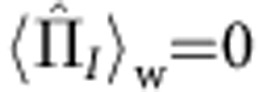
 and 
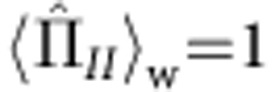
, the first of these expressions indicating that a weak interaction coupling the spatial wavefunction to a probe localized on path *I* has no effect on the probe on average, as if there was no neutron travelling on that path. Note that this is in accordance with the following theorem^18^: if the weak value of a dichotomic operator equals one of its eigenvalues, then the outcome of an ideal (also known as a strong) measurement of the operator is that same eigenvalue with probability one. So, if we were to weakly measure 
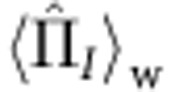
 and obtain 0, then, per the above theorem, since the projection operator is dichotomic and the weak value is an eigenvalue, we could also perform an ideal measurement of 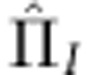 and again obtain 0. Similarly for 
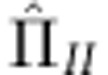
.

We can also rely on weak measurements to determine the location of the neutrons’ spin component. This is done by measuring the weak value of the spin component along each path *j* by applying a unitary interaction on the respective path. The appropriate observable to ascertain the weak value of the neutrons’ spin component on path *j* is 
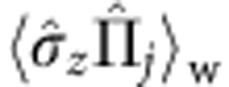
. The computation of the weak values yields 
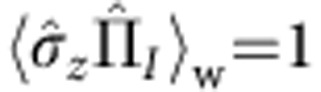
 and 
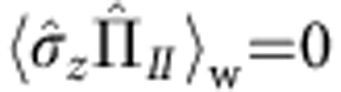
. On average, a weak interaction coupling with a probe on path *II* does not affect the state of that probe, as if there was effectively no spin component travelling along the path.

Note that in principle, it is possible to perform the weak measurements jointly along each path. We, however, carried them out separately (that is, in different runs). This is justified because a weak measurement made along a given path involves a local coupling, which in the weak limiting case only minimally disturbs the subsequent evolution of the quantum state along that path. It does not affect the evolution of the quantum state along the other path. This is in clear contrast with standard projective measurements, which would fundamentally disturb the system evolution by projecting the quantum state along both paths to a specific subspace. Then of course it would only be possible to make any inferences by appealing to counterfactual reasoning^25^.

### Experimental setup

**Figure 2:**
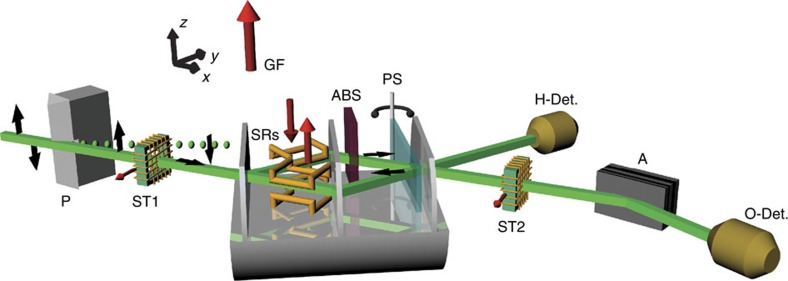
Illustration of the experimental setup. The neutron beam is polarized by passing through magnetic birefringent prisms (P). To prevent depolarization, a magnetic guide field (GF) is applied around the whole setup. A spin turner (ST1) rotates the neutron spin by *π*/2. Preselection of the system’s wavefunction 
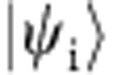
 is completed by two spin rotators (SRs) inside the neutron interferometer. These SRs are also used to perform the weak measurement of 
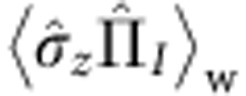
 and 
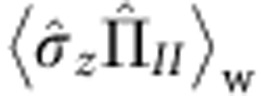
. The absorbers (ABS) are inserted in the beam paths when 
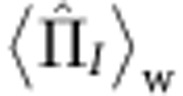
 and 
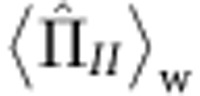
 are determined. The phase shifter (PS) makes it possible to tune the relative phase *χ* between the beams in path *I* and path *II*. The two outgoing beams of the interferometer are monitored by the H and O detector in reflected and forward directions, respectively. Only the neutrons reaching the O detector are affected by postselection using a spin turner (ST2) and a spin analyzer (A).

The experiment was performed at the S18 interferometer beam line at the research reactor of the Institut Laue-Langevin^32^. The setup is shown in Fig. 2.

A monochromatic neutron beam with a wavelength of *λ*=1.92 Å passes magnetic birefringent prisms, which polarize the neutron beam. To avoid depolarization, a magnetic guide field pointing in the +*z* direction is applied around the whole setup. A spin turner rotates the neutron spin by *π*/2 into the *xy* plane. The neutron’s spin wavefunction is then given by |*S*_*x*_;+›. Subsequently, the neutrons enter a triple-Laue interferometer^28^,^31^. Inside the interferometer, a spin rotator in each beam path allows the generation of the preselected state 
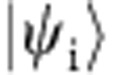
.

A phase shifter is inserted into the interferometer to tune the relative phase *χ* between path *I* and path *II*. Hence, a general postselected path state is given by 
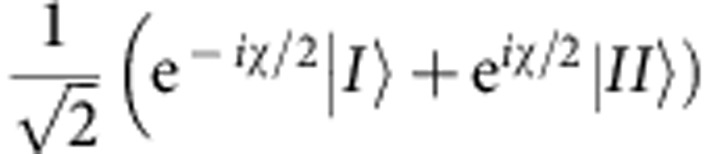
. Of the two outgoing beams of the interferometer, only the O beam is affected by a spin analysis. The H beam without a spin analysis is used as a reference monitor for phase and count-rate stability. The spin postselection of the O beam is done using a spin turner and a polarizing supermirror. Both outgoing beams are measured using ^3^He detectors with very high efficiency (over 99%). All measurements presented here are performed in a similar manner: The phase shifter is rotated, thereby scanning *χ* and recording interferograms. The interferograms allow to extract the intensity for *χ*=0. This ensures that the path postselection is indeed carried out on the state 
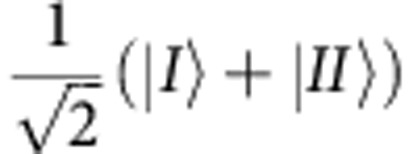
 corresponding to the O beam.

### Determining the neutrons’ population

**Figure 3:**
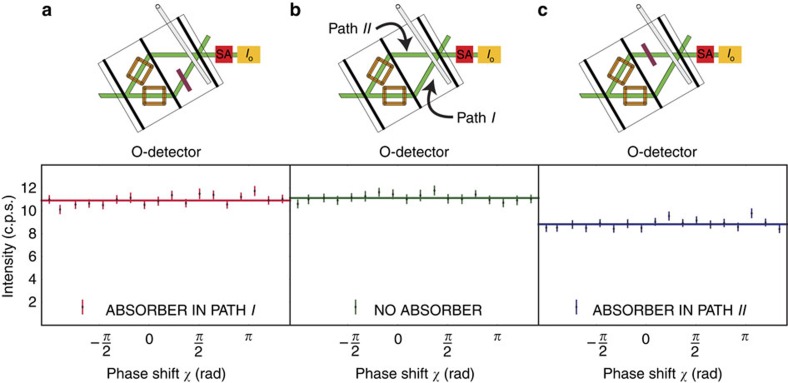
Measurement of 
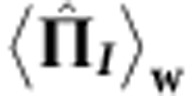
 and 
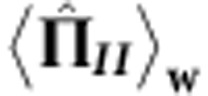
 using an absorber with transmissivity *T*=0.79(1). The intensity is plotted as a function of the relative phase *χ*. The solid lines represent least-square fits to the data and the error bars represent one s.d. (**a**) An absorber in path *I*; no significant loss in intensity is recorded. (**b**) A reference measurement without any absorber. (**c**) An absorber in path *II*: the intensity decreases. These results suggest that for the successfully postselected ensemble, the neutrons go through path *II*.

To determine the neutrons’ population in the interferometer’s paths, 
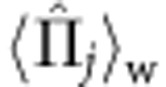
 are measured by inserting absorbers into the respective path *j* of the interferometer. 
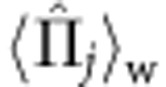
 is evaluated in three steps: first, a reference intensity is measured by performing a phase shifter scan of the empty interferometer to determine *I*^REF^. For the reference measurement, the spin states inside the interferometer are orthogonal. Therefore, the interferogram shows no intensity oscillation. As a second step, an absorber with known transmissivity of *T*=0.79(1) is inserted into path *I* and the phase shifter scan is repeated, which yields 
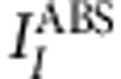
. Finally, the absorber is taken out of path *I* and put into path *II*. The subsequent phase shifter scan allows the extraction of 
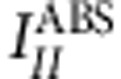
. A typical measurement result is depicted in Fig. 3.

In path *I*, the absorber has no effect. In comparison with the reference intensity, no significant change can be detected in the count rate. As opposed to this, the very same absorber decreases the intensity when it is put in path *II*. This already tells us that the neutrons’ population in the interferometer is obviously higher in path *II* than it is in path *I*.

### Determining the location of the neutrons’ spin component

**Figure 4:**
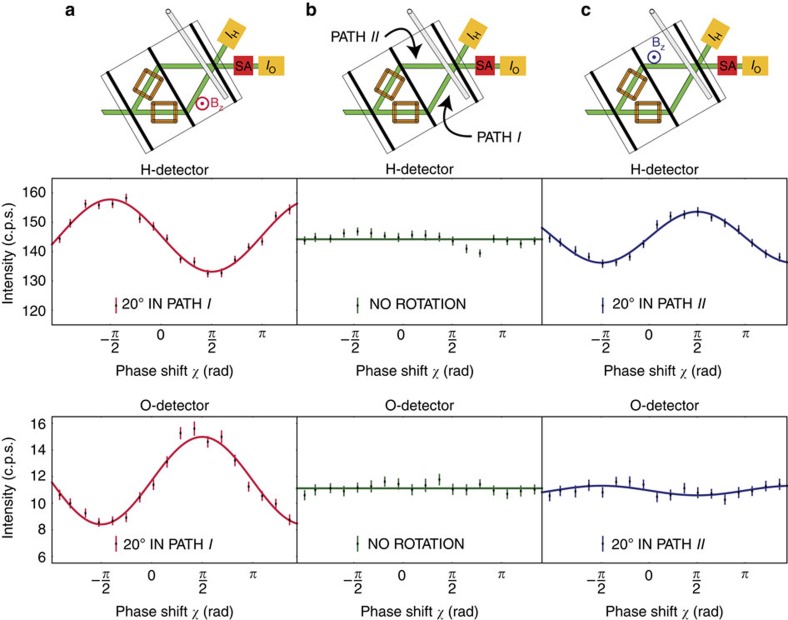
Measurement of 
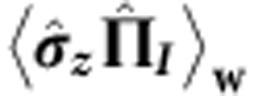
 and 
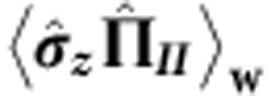
 applying small additional magnetic fields. The intensity of the O beam (with the spin analysis) and the H beam (without the spin analysis) is plotted as a function of the relative phase *χ*. The solid lines represent least-square fits to the data and the error bars represent one s.d. (**a**) A magnetic field in path *I*; interference fringes appear both at the postselected O detector and the H detector. (**b**) A reference measurement without any additional magnetic fields. Since the spin states inside the interferometer are orthogonal, interference fringes appear neither in the O, nor the H detector. (**c**) A magnetic field in path *II*; interference fringes with minimal contrast can be seen at the spin postselected O detector, whereas a clear sinusoidal intensity modulation is visible at the H detector without spin analysis. The measurements suggest that for the successfully postselected ensemble (only the O detector) the neutrons’ spin component travels along path *I*.

The weak measurements of the neutrons’ spin component in each path are achieved by applying additional magnetic fields in one or the other beam path. This causes a small spin rotation, which allows to probe the presence of the neutrons’ magnetic moment in the respective path. If there is a magnetic moment present in the path, the field has an effect on the measured interference fringes. If no change in the interferogram can be detected, there is no magnetic moment present in the path where the additional field is applied. The condition of a weak measurement is fulfilled by tuning the magnetic field small enough. A spin rotation of *α*=20° is applied. Using the correlation function, one can calculate the wavefunction overlap. It is 98.5%. The results of one such measurement procedure are shown in Fig. 4, where they are compared with the reference measurement performed with the empty interferometer.

Due to the orthogonal spin states inside the interferometer, the interferogram shows no intensity oscillation for both O and H detector (the contrast of a sinusoidal fit was 2.5% at most). An additional magnetic field in path *I* leads to the appearance of interference fringes at both O and H beam, giving 
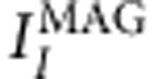
. The contrast of the O detector is 28.1%. Obviously, a magnetic moment is present in path *I*, since the interferogram’s contrast changes by an order of magnitude. Now the same field is applied in path *II* to obtain *I*_*II*_^MAG^. The field induces no significant change in the intensity modulation for the spin postselected O beam. The contrast of the O detector changes to only 3.8%. At the same time, a sinusoidal oscillation appears at the H beam, which has no spin analysis. If the ensemble is successfully postselected, the neutrons’ spin component travels along path *I*.

Figures 3 and 4 already clearly demonstrate the effect predicted by refs 23, 24, 25: an absorber with high transmissivity has on average no significant effect on the measurement outcome if it is placed in path *I*. It is only effective if it is placed in path *II*. In contrast to that, a small magnetic field has on average a significant effect only in path *I*, while it has none in path *II*. Therefore, any probe system that interacts with the Cheshire Cat system weakly enough will on average be affected as if the neutron and its spin are spatially separated.

### Weak values

We can now quantify this behaviour using weak values. To do so, intensities are extracted from the recorded interferograms. The intensities correspond to the value of the fit curve for *χ*=0. To gain precision, the measurements were repeated several times. Each experimental run allows extracting an intensity value from the data fit. The final results for the respective measurements are given by: *I*^REF^=11.25(5), 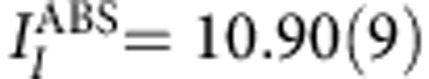, 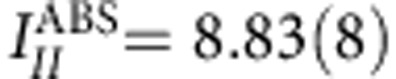, 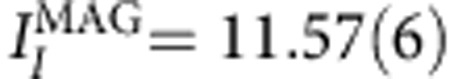 and 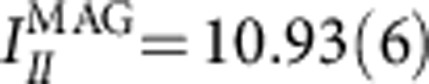 (all in counts per second). These intensities are the average of all performed measurements. Using these values, we obtain the results of the weak measurements (see Methods for details). They are: 
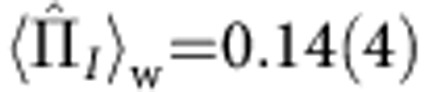
, 
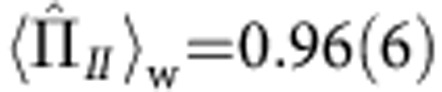
, 
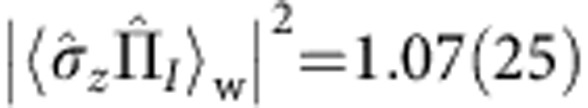
 and 
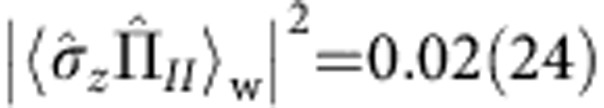
. Note that 
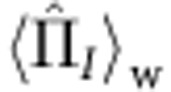
 and 
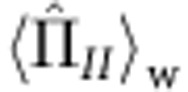
 must sum to unity: for the experimental values, we get 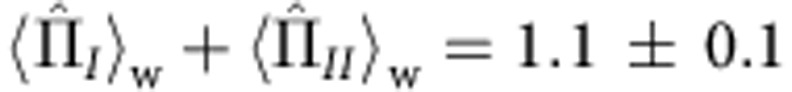, which confirms the theoretical predictions at the limit of error. The error in the determination of 
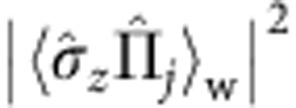
 is dominated by the statistical error of the count rate and by the systematic error that occurs during the spin manipulation.

Theory predicts that for the pre- and postselected states 
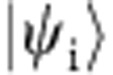
 and 
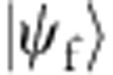
, the weak values for the spin components in paths *I* and *II* are given by 
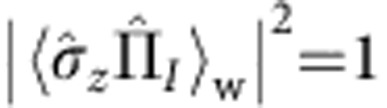
 and 
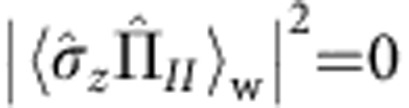
, while the weak values for the neutrons’ population along these paths are 
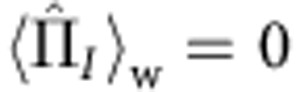
 and 
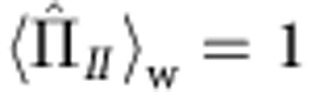
. Within the error, the experiment confirms this prediction. Slight deviations from the theoretical values were observed in the experiment: they manifest themselves in a minimal loss in intensity, when the absorber is inserted in path *I* and in the emergence of interference fringes with minimal contrast if a magnetic field is applied in path *II*. We suspect systematic misalignments in the experiment to cause this effect, mostly due to the finite degree of initial polarization, depolarization caused by the absorber and misalignment in the spin manipulation. These lead to an ‘imperfect separation’ of the particle and its property, which is reflected by a deviation of the weak values from 0–1.

## Discussion

Weak measurements combined with neutron interferometry allowed us to demonstrate a fundamental phenomenon of quantum mechanics, namely the quantum Cheshire Cat. It makes sense at the level of a pre- and postselected ensemble that a property of a quantum system can behave as being spatially separated from the site where one is certain to probe the particle's presence.

In general, it is true in quantum mechanics that one can give definite answers only for ensembles. So one has to bear in mind that, in general, no definite assertions about a single particle can be made. Indeed, there are many examples of situations which remain paradoxical from the perspective of individual particles^19^ but which were resolved at the level of ensembles^21^,^22^,^33^.

According to quantum theory, the wavefunction evolves through both paths of the interferometer. A weak interaction involving a coupling to the neutrons spatial wavefunction has on average no effect along path *I*, while a weak coupling involving the neutron spin wavefunction has observational consequences on average only when the coupling takes place along path *I* (ref. 24). Consequently, any probe system that interacts with the Cheshire Cat system weakly enough will on average be affected as if the neutron and its spin are spatially separated. The purpose of this article is to report an experimental observation of the quantum Cheshire Cat which was recently predicted theoretically^20^,^23^,^24^,^25^.

With respect to the interpretation of these empirical facts, there are many different approaches and perspectives. We briefly mention here a number of these perspectives.

One approach emphasizes that the weak value’s real part can be viewed as a conditioned average of the observable, reflecting the average value of the weakly measured observable given postselection^12^.

Another perspective does not interpret the weak value as being a real property of the system, but as an optimal estimate of the corresponding observable, given that the postselection is successful. Then it can be argued that the observable has no definite value between pre- and postselection, and the real part of the weak value can be connected to the Bayes estimator of the observable on a pre and postselected ensemble^34^.

Finally, we want to emphasize that the weak value is completely general: any weak coupling will result in a shift of the measuring device by the relevant weak value. The Cheshire Cat phenomenon is also completely general and can be applied to any quantum object. These qualities therefore open the possibility for future applications of the quantum Cheshire Cat such as high-precision metrology and quantum information technology^12^,^16^,^25^. For example, one could imagine a situation in which the magnetic moment of a particle overshadows another of the particle’s properties which one wants to measure very precisely. The Cheshire Cat effect might lead to a technology which allows one to separate the unwanted magnetic moment to a region where it causes no disturbance to the high-precision measurement of the other property.

## Methods

### Weak measurement of 
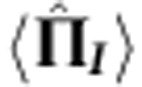
 and 
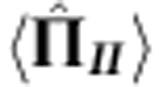


The weak measurement of 
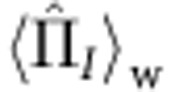
 and 
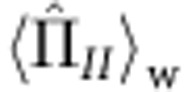
 is performed using absorbers with a high transmissivity (that is, a ‘weak absorption’). Phenomenologically, an absorption in path *j* can be represented by an imaginary optical potential:


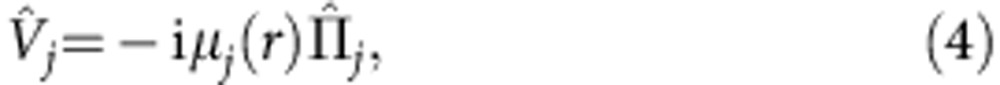


where the absorption coefficient is given by *M*_*j*_=∫*μ*_*j*_(*r*)d*r* in path *j* with *r* integrated on the absorber slab thickness. For weak absorption, *M*_*j*_ can be related to the transmissivity *T*_*j*_ through *M*_*j*_≈1−(*T*_*j*_)^1/2^ (ref. 35). For simplicity the free evolution operator is omitted in the following expressions. The wavefunction after the wavepacket has interacted with the absorber along path *j* is


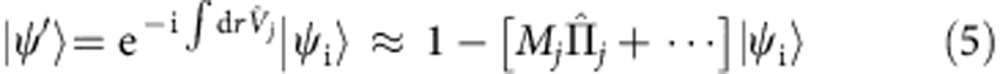


for small *M*_*j*_. Using 
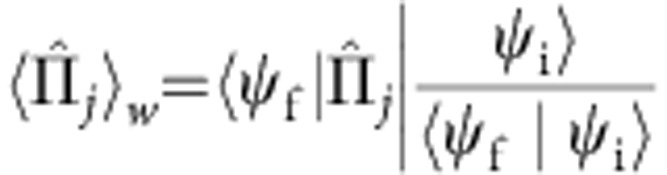
, the intensity for the postselected outcome takes the form


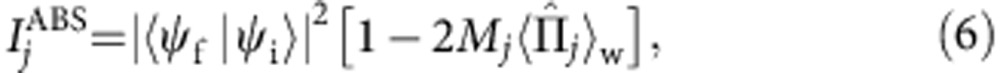


where the assumption was made that the contribution of the imaginary part of 
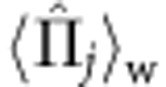
 is of order or less then *M*^2^. This is justified, if the experimental pre- and postselected states are indeed given by equations (2) and (3). Experimentally, this can be checked by the contrast of the empty interferometer *C*, since any deviation from the ideal pre- and postselected state would manifest itself in the emergence of interference fringes. However, in the experiment, the average maximal contrast was only *C*≤0.024(5) confirming the aforementioned assumptions. Since 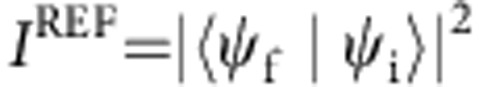, the weak values can be extracted from the observation of 
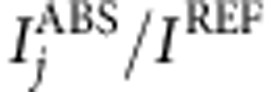
.

### Weak measurement of 
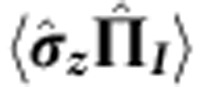
 and 
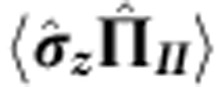


The weak value of 
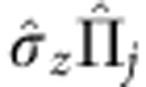
 is determined using path-conditioned spin rotations. To measure 
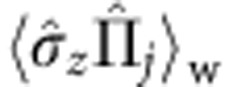
, a small magnetic field is applied in path *j*. The interaction Hamiltonian for this measurement is


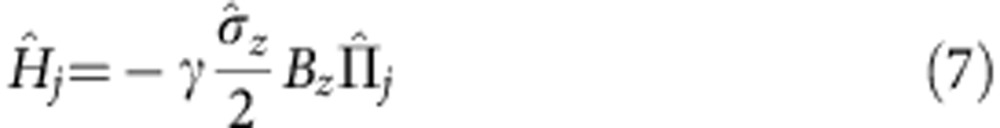


where *γ* is the gyromagnetic ratio and *B*_*z*_ is an externally applied magnetic field. 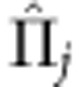 denotes the fact that the magnetic field is applied only in the region along path *j*. Applying a magnetic field along *z* leads to the 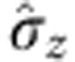 component of the Pauli matrix, which is the generator of rotations around the *z* axis. Thus, a small rotation around *z* on path *j* is generated through the coupling between the magnetic field and the spin projection of the neutron on the *z* axis. The rotation angle produced by this Larmor precession effect will be labelled by *α*; its magnitude is proportional to the magnetic field strength^28^. The evolution of the initial state caused by the weak measurement is given by


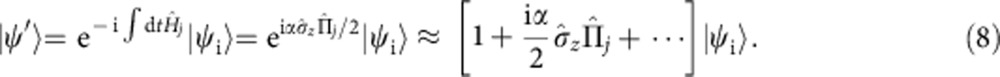


After postselection for the outcomes corresponding to the final state 
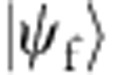
, the intensity at the O detector is


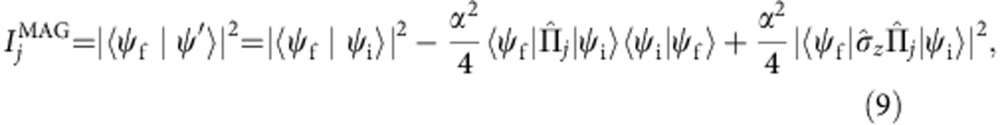


taking into account *α* up to *α*^2^. As in the derivation of the relation to extract 
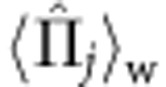
, the assumption is made that the imaginary part of 
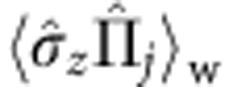
 is of order or less then *α*^3^. Again this is justified by the average maximal contrast of the empty interferometer. Consequently we get:


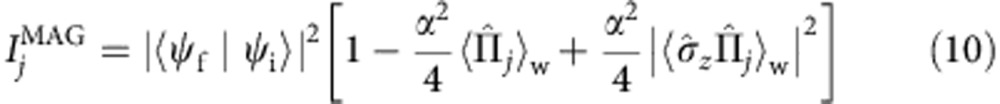


Since 
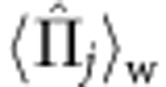
 is known from the absorber measurements and 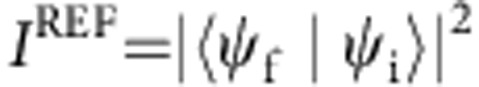, the weak values are extracted from the measurements of the intensities with the magnetic field along path *I*, along path *II* and with the magnetic field turned off.

### Data treatment

When the pre- and postselected states 
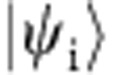
 and 
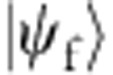
 are given by


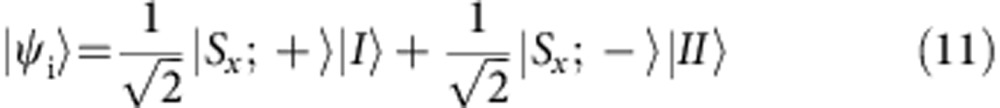


and


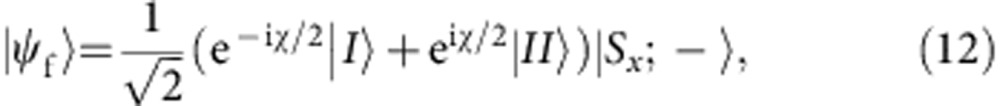


one can easily see that for an ideal system, the normalized intensities for the O detector are


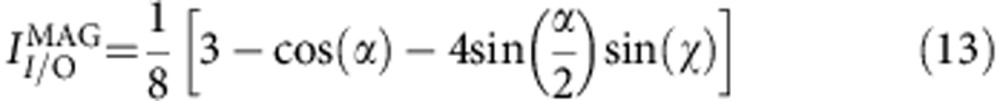


and


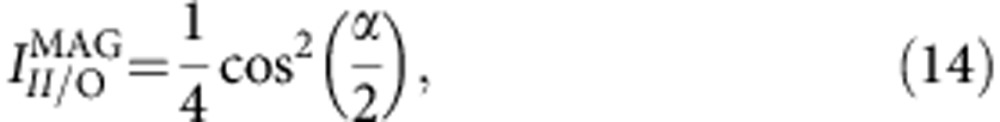


if a rotation of angle *α* is applied in the respective paths. Since the H detector is not spin analysed, the expected normalized intensities are simply given by


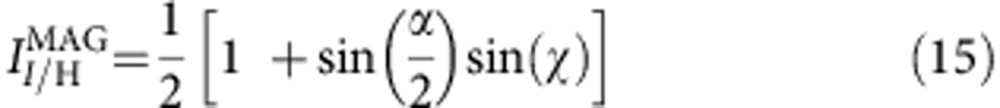


And


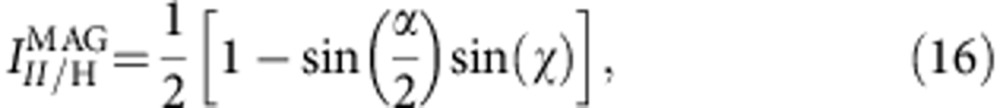


again if a rotation of angle *α* is applied in the respective paths and for an ideal system.

Due to the orthogonal preselected spin state, no oscillation fringes appear for *α*=0, that is, for the reference intensity. The experimental results are in very good agreement with the theoretical calculations. The reference measurements show an average contrast of *C*≤0.024(5), while the maximal contrast achieved with the neutron interferometer used for the experiment is *C*≈0.85. Therefore, the reference intensity *I*^REF^ is fitted with a line.

When a spin rotation is applied along path *I*, an intensity oscillation dependent on the relative phase *χ* is expected to appear at both the O and H detector. Hence, the data are fitted with a sine curve of the form


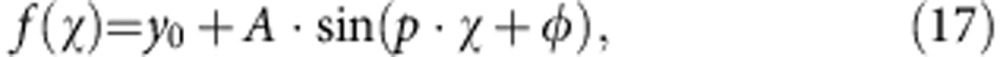


where *y*_0_ is the offset, *A* is the amplitude, *p* is the period and *φ* the phase of the fit function. Since the H detector is not spin analysed, its count rate is much higher and it offers much better statistics. The period of the fit is determined from the H detector measurement and then fixed for the O detector fit. In addition to that, the H detector is used to normalize the O detector intensity for count-rate stability. The intensity 
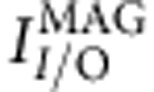
 is then taken from the fit of the O detector data for *χ*=0.

If a spin rotation is applied along path *II*, no intensity oscillation is expected at the O detector, while interference fringes should appear at the H detector. As predicted, 
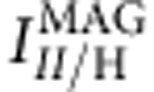
 shows clear sinusoidal oscillations, with a contrast similar to the 
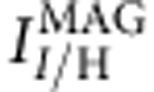
 measurements. However, due to systematic misalignments in the experiment, the most apparent one being the finite degree of initial polarization, depolarization caused by the absorber and misalignment in the spin manipulation, interference fringes with minimal contrast can be detected also for 
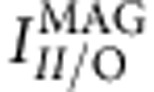
. Therefore, the data for 
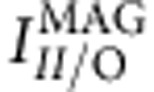
 is also fitted with a sine curve using a fixed period determined by the H detector. Again the intensity 
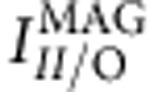
 is taken from the fit of the O detector data for *χ*=0.

To gain precision, several phase shifter scans were performed to measure *I*^REF^ and 
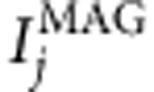
. The data of each phase shifter scan are then fitted and the intensity is taken from the fit for *χ*=0. The average of all measurements performed is then used to calculate the weak values. The same reference intensity is used to normalize the path and the spin measurements.

## Author contributions

T.D. and Y.H. conceived the experiment; T.D., H.G., S.S. and Y.H. designed the experiment and analysed the data; T.D., H.G., S.S. and H.L. carried out the experiment; A.M. and J.T. supplied the theoretical part; all authors co-wrote the paper.

## Additional information

**How to cite this article:** Denkmayr, T. *et al.* Observation of a quantum Cheshire Cat in a matter-wave interferometer experiment. *Nat. Commun.* 5:4492 doi: 10.1038/ncomms5492 (2014).

## Figures and Tables

**Figure 1:**
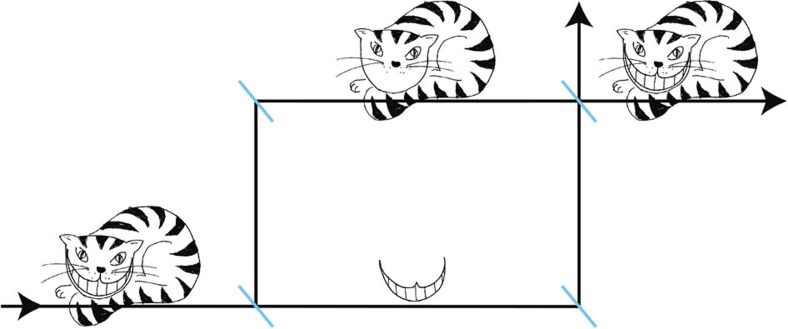
Artistic depiction of the quantum Cheshire Cat. Inside the interferometer, the Cat goes through the upper beam path, while its grin travels along the lower beam path. Figure courtesy of Leon Filter.
